# MiR-497-5p Regulates Osteo/Odontogenic Differentiation of Stem Cells From Apical Papilla via the Smad Signaling Pathway by Targeting *Smurf2*

**DOI:** 10.3389/fgene.2020.582366

**Published:** 2020-10-30

**Authors:** Junqing Liu, Xiaolong Wang, Mengxiao Song, Jing Du, Jiali Yu, Wenzhou Zheng, Chengfei Zhang, Yan Wang

**Affiliations:** ^1^Department of Vip center, School and Hospital of Stomatology, Cheeloo College of Medicine, Shandong University & Shandong Key Laboratory of Oral Tissue Regeneration & Shandong Engineering Laboratory for Dental Materials and Oral Tissue Regeneration, Jinan, China; ^2^Endodontics, Faculty of Dentistry, The University of Hong Kong, Hong Kong, Hong Kong; ^3^Department of Breast Surgery, Qilu Hospital of Shandong University, Jinan, China; ^4^Department of Stomatology, The First Affiliated Hospital of Zhengzhou University, Zhengzhou, China; ^5^Department of Oral Pathology, School of Stomatology, Zhengzhou University, Zhengzhou, China

**Keywords:** miR-497-5p, stem cells from apical papilla, Smurf2, Smad signaling pathway, osteo/odontogenic differentiation

## Abstract

Osteo/odontogenic differentiation is a key process of human stem cells from apical papilla (SCAP) in tooth root development. Emerging evidence indicates microRNAs (miRNAs) play diverse roles in osteogenesis. However, their functions in osteo/odontogenic differentiation of SCAP require further elucidation. To investigate the role of miRNA in SCAP osteo/odontogenic differentiation and underlying mechanisms, miRNA microarray analysis was performed to screen differentially expressed miRNAs between control and osteo/odontogenic-induced group. Quantitative real-time PCR (qRT-PCR) and western blot were used to detected osteo/odontogenic differentiation-related markers and possible signaling pathway SCAP-associated genes. Alizarin Red Staining (ARS) were applied to evaluated osteogenic capacity. The results showed that miR-497-5p increased during SCAP osteo/odontogenic differentiation. Overexpression of miR-497-5p enhanced the osteo/odontogenic differentiation of SCAP, whereas downregulation of miR-497-5p elicited the opposite effect, thus suggesting that miR-497-5p is a positive regulator of the osteo/odontogenic differentiation of SCAP. Bioinformatic analysis and dual luciferase reporter assay identified that SMAD specific E3 ubiquitin protein ligase 2 (*Smurf2*) is a direct target of miR-497-5p. Further study demonstrated that *Smurf2* negatively regulates SCAP osteo/odontogenic differentiation, and silencing *Smurf2* could block the inhibitory effect of the miR-497-5p inhibitor. Meanwhile, pathway detection manifested that miR-497-5p promotes osteo/odontogenic differentiation via Smad signaling pathway. Collectively, our findings demostrate that miR-497-5p promotes osteo/odontogenic differentiation of SCAP via Smad signaling pathway by targeting *Smurf2*.

## Introduction

Stem cells from apical papilla (SCAP) were first isolated and characterized from the apical papilla of human immature third molar by Sonoyama et al. (2006, 2008). This indicates that SCAP play a vital role in the development of tooth root. Similar to postnatal mesenchymal stem cells (MSCs), SCAP possess self-renewal and multidirectional differentiation potential (Abe et al., 2008; Sonoyama et al., 2008; Bakopoulou et al., 2011; Koutsoumparis et al., 2018). Compared with dental pulp stem cells (DPSCs), SCAP have stronger proliferation ability, cell migration, and telomere activity, and are considered to be a valuable source of postnatal MSCs for dental tissue engineering (Sonoyama et al., 2006; Huang et al., 2010; Na et al., 2016; Chrepa et al., 2017). The osteo/odontogenic differentiation of SCAP is prerequisite in pulp-dentine regeneration. Therefore, how to effectively promote the osteogenic/odontogenic differentiation of SCAP has become a key issue. Our previous studies have demonstrated that many factors could regulate osteo/odontogenic differentiation of SCAP (Yang et al., 2012; Wan et al., 2016; Liu et al., 2019a). However, the molecular mechanisms underlying the osteo/odontogenic differentiation in SCAP remains unknown.

MicroRNAs (miRNAs) are a class of small single-stranded non-coding RNAs. They function as post-transcriptional regulators. MiRNAs bind to 3′-untranslated regions (3′-UTR) of the target mRNA and modulate the expression of target genes through degrading target mRNAs or inhibiting their translation. MiRNAs have been implicated in various biological processes, including cell proliferation, differentiation, apoptosis, and carcinogenesis (Hammond et al., 2001; Bartel, 2004; Lindsay, 2008; Julia et al., 2009). A number of miRNAs were reported to regulate osteogenic differentiation in mesenchymal stem cells (Chen et al., 2017; Tang et al., 2018). Accumulating evidence revealed that miRNAs participated in osteogenic differentiation of dental stem cells. MiR-214 down-regulated the osteogenic differentiation of periodontal ligament stem cells (PDLSCs) by targeting transcription factor 4 (Yao et al., 2017). MiR-508-5p suppressed the osteogenesis of human dental pulp stem cells through inhibiting glycoprotein non-metastatic melanoma protein B (Liu et al., 2019b). NOTCH activation inhibited osteogenic differentiation of SCAP and promoted the expression of miR-34a, while miR-34a suppressed Notch signaling by targeting *NOTCH2* and *HES1* (Sun et al., 2014). To date, there have been few studies on miRNA regulating osteo/odontogenic differentiation of SCAP and their molecular mechanisms remained still unclear. Thus, we hypothesized that certain miRNAs could positively regulate the osteo/odontogenic differentiation of SCAP, which could be used as a novel target for regulating dental tissue regeneration.

In this study, a microarray was applied to investigate the miRNA expression profiles of SCAP during osteo/odontogenic differentiation. qRT-PCR was performed to verify gene expression. Bioinformatics and qRT-PCR analysis demonstrated miR-497-5p was up-regulated during osteogenic differentiation. Further study confirmed that miR-497-5p increased the osteo/odontogenic differentiation of SCAP. Target gene prediction and dual luciferase reporter assay showed that *Smurf2*, a negative regulator of osteogenesis, was a direct target of miR-497-5p. The Smad signaling pathway was involved in the osteo/odontogenic differentiation of SCAP. Our study suggested miR-497-5p could suppress *Smurf2* and promote osteo/odontogenic differentiation of SCAP through Smad signaling pathway.

## Materials and Methods

### Cell Culture

Human apical papilla tissues were obtained from immature third permanent molars extracted from patients aged 16–20 years for orthodontic reason at the School and Hospital of Stomatology, Shandong University. This project was approved by the Ethics Committee of the School and Hospital of Stomatology, Shandong University. The consent was obtained from the patients or their parents. The apical papilla was separated from the third molar and digested with 3 mg/mL Collagenase Type I (Solarbio, Beijing, China) and 4 mg/mL Dispase (Roche, Indianapolis, IN, United States) at 37°C for 1 h. Then cells were cultured in α-minimum essential medium (HyClone, Logan, UT, United States) supplemented with 20% fetal bovine serum (FBS) (Gibco, Grand Island, NY, United States), 100 U/mL penicillin and 100 μg/mL streptomycin (HyClone) incubated at 37°C in a 5% CO_2_ atmosphere. The cells from passage 2–5 were used for subsequent experiments.

Human embryonic kidney 293T (HEK 293T) cells were cultured in DMEM medium containing 10% FBS, 100 U/ml penicillin and 100 μg/ml streptomycin.

### Flow Cytometry

SCAP were characterized by flow cytometry. A total of 8 × 10^5^ cells were collected and incubated respectively with monoclonal antibodies specific for STRO-1 (R&D Systems, Minneapolis, MN, United States), CD24, CD146, CD34 and CD45 (BD Biosciences, Franklin Lakes, NJ, United States) for 30 min at room temperature. Expression profiles were analyzed by BD FACSCalibur flow cytometer (BD Biosciences).

### Cell Counting Kit-8 (CCK-8) Analysis

The proliferation ability of SCAP was examined using CCK-8 (MedChemExpress, Monmouth Junction, NJ, United States). Cells were seeded in 96-well plates at a density of 3 × 10^3^ cells/well. The cells were treated with CCK-8 reagent on days 1, 2, 3, 4, 5, 6, 7, 8, 9, and 10 and incubated at 37°C for 2 h. Then, the absorbance was measured at 450 nm using a microplate reader.

### Osteogenic Differentiation

For osteogenic differentiation, after the SCAP reached 80% confluence, the medium was changed to osteogenic medium containing 10 mM β-glycerolphosphate (Sigma-Aldrich, St. Louis, MO, United States), 10 nM dexamethasone (Sigma-Aldrich) and 50 μg/mL L-ascorbic acid (Sigma-Aldrich). The osteogenic medium was changed every 3 days.

### Alkaline Phosphatase (ALP) Activity Assay

SCAP were cultured in osteogenic medium for 7 days. Then cells were washed with PBS and lysed with radioimmunoprecipitation (RIPA) lysis buffer (Solarbio) on ice for 30 min. After sonication and centrifugation, the supernatants were obtained and ALP activity was measured using an ALP activity assay kit (Jiancheng, Nanjing, China), according to the manufacturer’s instructions.

### ALP Staining and Alizarin Red Staining (ARS)

SCAP were grown in osteogenic medium. On day 7, cells were fixed with 4% paraformaldehyde, followed by washing with PBS. ALP staining was performed using an ALP staining kit (Solarbio), according to manufacturer’s protocol. For Alizarin Red staining, after 21 days induction, cells were fixed with 4% paraformaldehyde and stained with 2% Alizarin red (Sigma-Aldrich) with pH 4.2 for 30 min. Then, cells were rinsed thrice with double distilled water to remove the unbound dye and visualized under a microscope. For quantitative assay, the alizarin red was dissolved in 10% cetylpyridinium chloride (Sigma-Aldrich) for 30 min. The absorbance was detected at 560 nm and the results were normalized to the total protein content.

### MiRNA Microarray and Identification of Differentially Expressed Genes

SCAP treated with osteogenic induction for 7 days were served as experimental group (*n* = 3), while the untreated cells were control group (*n* = 3). Total RNA was extracted and analyzed miRNAs expression using microarray (Affymetrix miRNA 4.0 Array). To determine differentially expressed genes, the R package “limma” was used (Ritchie et al., 2015). MiRNAs (fold change ≥2.0 or ≤0.5, *P* value <0.05, compared with control group) were considered to be statistically significant. The corresponding heatmaps were drawn by R package “pheatmap” (Wang et al., 2019).

### Cell Transfection

Synthetic miR-497-5p mimics/inhibitor, siRNA targeting *Smurf2* (siSmurf2), and their negative control (NC) were purchased from GenePharma. SCAP were transfected with these oligonucleotides using Lipofectamine^TM^ 2000 (Invitrogen, Carlsbad, CA, United States) following the manufacturer’s instructions. For osteo/odontogenic induction, the medium was changed to osteogenic medium after 24 h transfection.

### Dual Luciferase Reporter Assay

TargetScan, miRPathDB, starBase and PiTA database were used to predict potential targets of miR-497-5p. The dual luciferase reporter assay was performed to determine whether miR-497-5p could directly regulate *Smurf2*. A Smurf2 3′-UTR reporter vector was synthetized by BioSune Biotechnology. The wild type (WT) and mutant-type (Mut) plasmid was termed Smurf2-WT and Smurf2-Mut, respectively. HEK293T cells were cultured in a 96-well plate. Then Smurf2-WT or Smurf2-Mut was co-transfected with miR-497-5p mimics or its negative control into HEK293T cells. After 48 h of incubation, firefly and renilla luciferase activities were analyzed using the Dual-Luciferase Reporter Assay System (Promega, Madison, WI, United States). Renilla luciferase activity was used as an internal control.

### Quantitative Real-Time PCR (qRT-PCR) Analysis

Total RNA was extracted from osteogenic induced cells using RNAiso Plus (Takara, Shiga, Japan). Complementary DNA (cDNA) was synthesized with a Reverse Transcription System (Takara) following the manufacturer’s protocol. qRT-PCR was performed on by Roche LightCycler^§^ 480 (Roche, Mannheim, Germany) using SYBR Premix Ex Taq (Takara). Relative expression of mRNA or miRNA were evaluated, GAPDH or U6 was used as an endogenous normalization control. The primers used for amplification were listed in the Supplementary Table 1.

### Western Blot Analysis

After 7 days of osteogenic induction, the transfected SCAP were harvested and lysed with RIPA lysis buffer containing 1% phenylmethylsulfonyl fluoride (PMSF) (Beyotime, Shanghai, China) on ice. Total protein concentrations were measured by the BCA Protein Assay Kit (Solarbio). The proteins in all samples were separated by SDS-PAGE gels, and transferred to polyvinylidene fluoride (PVDF) membranes (Millipore, Billerica, MA, United States). After blocking in 5% skim milk for 1 h at room temperature, the membranes were incubated with primary antibodies overnight at 4°C. Primary antibodies included DSPP (Santa Cruz Biotechnology, Santa Cruz, CA, United States), Collagen I (Wanlei, Shenyang, China), ALP (Abcam, Cambridge, MA, United Kingdom), Runx2, OSX (Abcam), OPN (Abcam), SNIP1 (Abcam), Smurf1 (Proteintech Group, Chicago, IL, United States), Smurf2, Smad2, Smad3, Smad4 (Cell Signaling Technology), and GAPDH (Proteintech Group). After that, the membranes were incubated with horseradish peroxidase–conjugated anti-mouse or anti-rabbit secondary antibodies (Proteintech Group) at room temperature for 1 h. Protein bands were visualized using the enhanced chemiluminescence (Millipore) and the protein levels were quantified using the ImageJ software.

### Statistical Analysis

Data are presented as the mean ± SD from at least three independent experiments. The t-test, one-way and two-way ANOVA were used for statistical analysis. *P* < 0.05 was considered statistically significant. All statistical analyses were performed using the GraphPad Prism 6.

## Results

### Characteristics of SCAP and Osteogenic Differentiation

**FIGURE 1:**
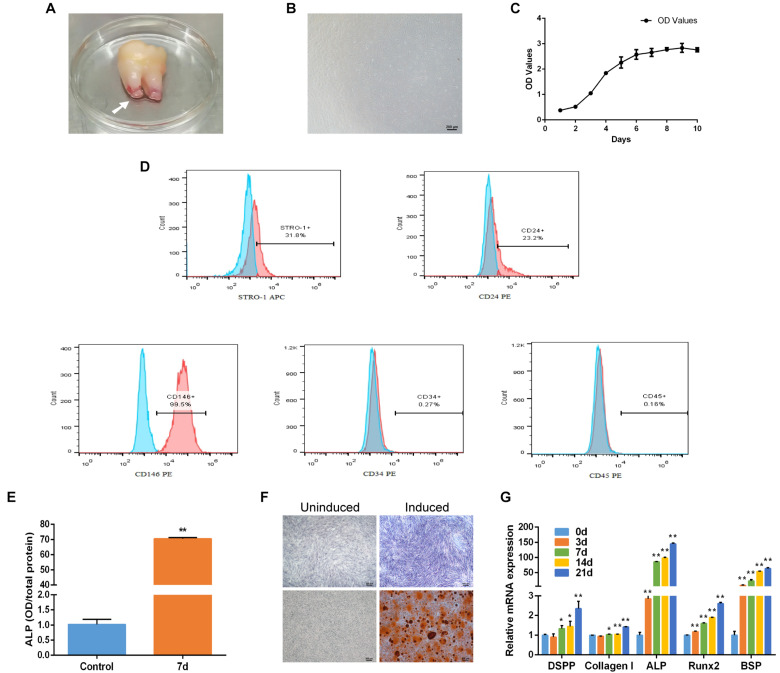
Characteristics and osteogenic differentiation of SCAP. **(A)** Human apical papilla (white arrow) attached to the apex of extracted immature third molar. **(B)** The morphology of SCAP. **(C)** Growth curve gained according to CCK-8 method. **(D)** Cell surface markers of SCAP displayed by flow cytometry. **(E)** ALP activity at day 7 of osteogenic differentiation. **(F)** ALP staining on day 7 and Alizarin red staining on day 21 after osteogenic induction. **(G)** Osteogenic specific genes examined by qRT-PCR at different time points. Data are presented as mean ± SD of three independent experiments. *P < 0.05, **P < 0.01 compared with control group.

Human SCAP was isolated and adhered to the culture dish with spindle-liked morphology (Figures 1A,B). CCK-8 assayed the proliferation of SCAP including the incubation, logarithmic growth, and plateau periods. The growth curve was shown in Figure 1C. Flow cytometry analysis showed that cell surface markers of SCAP were positive for STRO-1 (31.8%), CD24 (23.2%) and CD146 (99.5%), and negative for CD34 (0.27%) and CD45 (0.16%) (Figure 1D). After osteogenic induction, the ALP activity increased significantly compared with control group (Figure 1E). ALP staining and Alizarin red staining showed a clear bluish violet stains and mineralized nodules, respectively (Figure 1F). The mRNA expression of specific markers, including Dentin sialophosphoprotein (*DSPP*), Collagen type I (*Collagen I*), Alkaline phosphatase (*ALP*), Runt-related transcription factor 2 (*Runx2*) and Bone sialoprotein (*BSP*), were upregulated after the osteogenic induction (Figure 1G). These results revealed that SCAP has a good proliferation ability and osteogenic differentiation potential.

### Increased Expression of miR-497-5p During Osteo/Odontogenic Differentiation of SCAP

**FIGURE 2:**
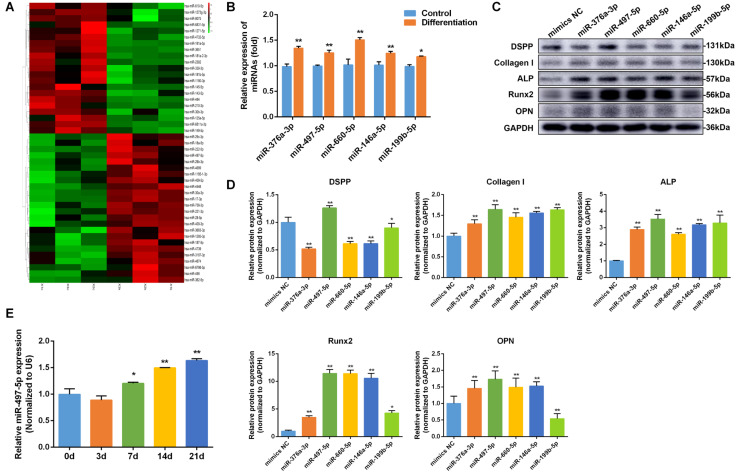
miR-497-5p was upregulated during osteo/odontogenic differentiation of SCAP. **(A)** Heat map of the differentially expressed miRNAs in differentiated SCAP. Red indicates up-regulated miRNAs and green indicates down-regulated miRNAs. **(B)** Expression levels of miR-376a-3p, miR-497-5p, miR-660-5p, miR-146a-5p, miR-199b-5p in differentiated SCAP examined by qRT-PCR. **(C,D)** The expression of osteo/odontogenic differentiation related proteins in overexpressed miR-376a-3p, miR-497-5p, miR-660-5p, miR-146a-5p, miR-199b-5p examined by western blot. **(E)** Time course of miR-497-5p expression during osteogenic differentiation of SCAP. Data are presented as mean ± SD of three independent experiments. *P < 0.05, **P < 0.01 compared with control group.

MiRNA microarray analysis showed that miRNAs differentially expressed between osteo/odontogenic induction SCAP and control group. Heatmap revealed that the expression levels of 45 miRNAs significantly changed, with 24 miRNAs up-regulated and 21 down-regulated compared to those in the control group (Figure 2A). To verify the microarray data, five miRNAs from differentially expressed miRNAs were selected and detected using qRT-PCR. The results showed that all five miRNAs were significantly changed and were consistent with microarray data (Figure 2B). In order to screen for the most differentially expressed miRNA in osteo/odontogenic differentiation, five miRNAs were overexpressed by transfecting miRNAs mimics and the protein expression of osteo/odontogenic differentiation-related genes (*DSPP*, *Collagen I*, *ALP*, *Runx2*, *and OPN*) was detected using western blot. We found these markers were increased significantly in miR-497-5p group compared to those in the NC group (Figures 2C,D). To further observe the phase changes of miR-497-5p in the SCAP osteo/odontogenic differentiation, we determined its expression and found that miR-497-5p increased on the day 7 and remained high level until day 21 (Figure 2E), indicating that miR-497-5p might promote the osteo/odontogenic differentiation of SCAP.

### MiR-497-5p Promoted Osteo/Odontogenic Differentiation in SCAP

**FIGURE 3:**
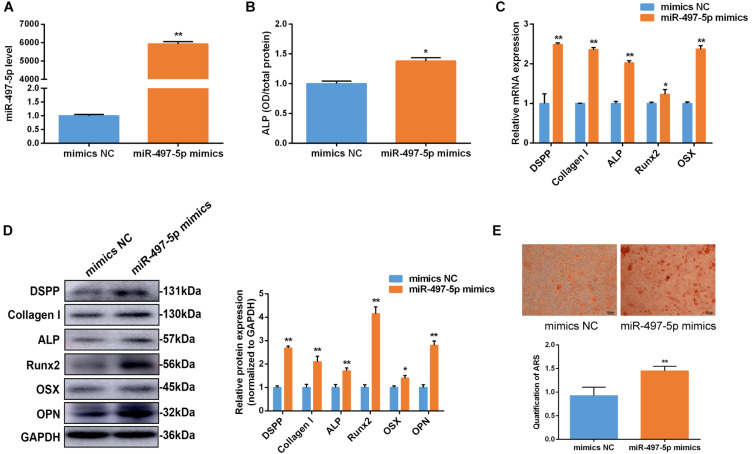
miR-497-5p promoted osteo/odontogenic differentiation in SCAP. **(A)** miR-497-5p expression assessed by qRT-PCR in SCAP transfected with miR-497-5p mimics. **(B)** ALP activity measured on day 7 of osteogenic differentiation. **(C,D)** mRNA and protein expression of osteo/odontogenic markers after miR-497-5p overexpression. **(E)** Alizarin red staining and quantitative assay after miR-497-5p overexpression. Data are presented as mean ± SD of three independent experiments. *P < 0.05, **P < 0.01 compared with control group.

To determine whether miR-497-5p regulated osteo/odontogenic differentiation, miR-497-5p mimics was transfected into SCAP and the cells were cultured in osteogenic medium. The levels of miR-497-5p were elevated by approximately 6,000-fold compared to those in the NC group, as confirmed by qRT-PCR after 24 h of transfection, which indicated high transfection efficiency (Figure 3A). The overexpression of miR-497-5p enhanced the ALP activity and the mRNA expression of osteo/odontogenic specific genes, including *DSPP*, *Collagen I*, *ALP*, *Runx2* and Osterix (*OSX*), compared to that of negative control (Figures 3B,C). Consistently, the protein levels of DSPP, Collagen I, ALP, Runx2, OSX, OPN were upregulated in miR-497-5p overexpression group (Figure 3D). The Alizarin red staining also showed enhanced osteogenic capacity of SCAP (Figure 3E). These findings suggested that miR-497-5p might be a promoter for osteo/odontogenic differentiation of SCAP.

### Inhibition of miR-497-5p Suppressed Osteo/Odontogenic Differentiation in SCAP

**FIGURE 4:**
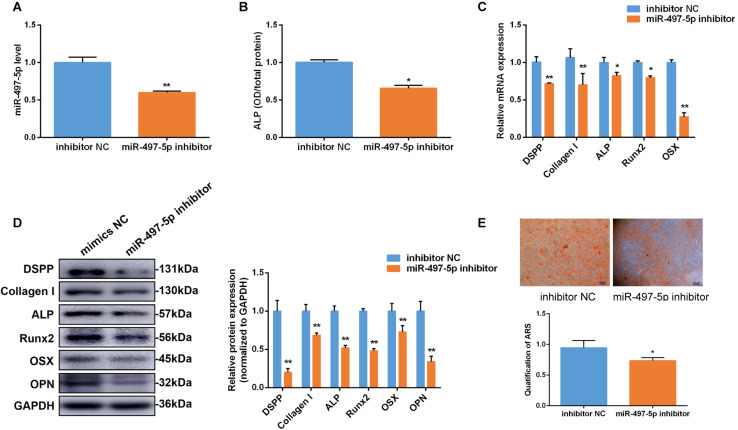
Inhibition of miR-497-5p suppress osteo/odontogenic differentiation in SCAP. **(A)** miR-497-5p expression assessed by qRT-PCR in SCAP transfected with miR-497-5p inhibitor. **(B)** ALP activity measured on day 7 of osteogenic differentiation. **(C,D)** mRNA and protein expression of osteo/odontogenic markers examined after miR-497-5p suppression. **(E)** Alizarin red staining and quantitative assay after miR-497-5p suppression. Data are presented as mean ± SD of three independent experiments. *P < 0.05, **P < 0.01 compared with control group.

To further clarify the effect of miR-497-5p on the osteo/odontogenic differentiation, miR-497-5p inhibitor was transfected into SCAP and the cells were cultured in osteogenic medium. The inhibition efficiency of miR-497-5p was detected (Figure 4A). Consequently, the ALP activity was suppressed (Figure 4B). The osteo/odontogenic specific markers (*DSPP*, *Collagen I*, *ALP*, *Runx2*, *OSX*, and *OPN*) were remarkably down-regulated compared to their negative controls (Figures 4C,D). The Alizarin red staining reflected the same effects (Figure 4E). All the results thus suggest that down-regulating miR-497-5p could suppress the osteo/odontogenic differentiation of SCAP.

### miR-497-5p Directly Targeted *Smurf2*

**FIGURE 5:**
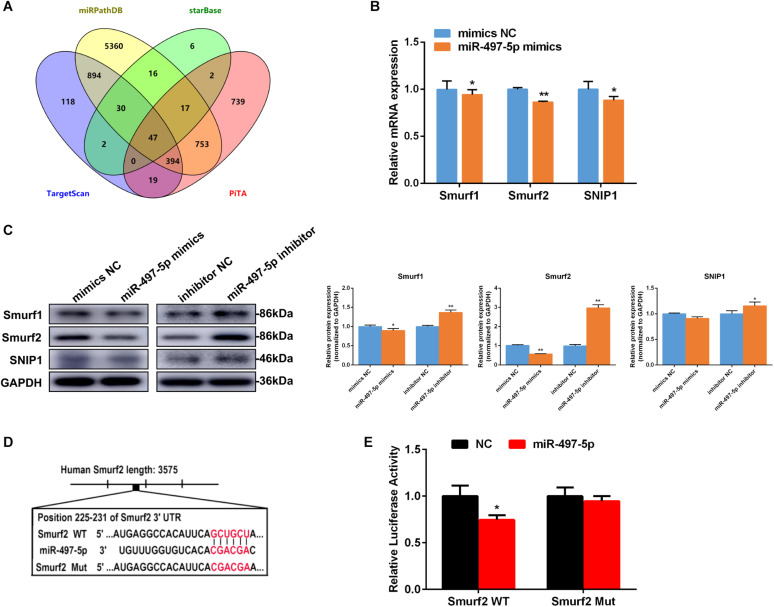
miR-497-5p directly targets *Smurf2*. **(A)** Venn diagram showed number of miR-497-5p target genes predicted by performing TargetScan, miRPathDB, starBase and PiTA. **(B,C)** Candidate gene expressions in SCAP detected by qRT-PCR and western blot after transfected with the miR-497-5p mimics/inhibitor. **(D)** Schematic diagrams indicating the Smurf2 wild-type (WT) contains miR-497-5p binding sites. **(E)** Luciferase activities of Smurf2 3′UTR WT or Mut reporter plasmids. Data are presented as mean ± SD of three independent experiments. *P < 0.05, **P < 0.01 compared with control group.

To reveal the probable molecular mechanism by which miR-497-5p mediates osteo/odontogenic differentiation of SCAP, TargetScan, miRPathDB, starBase and PiTA were used to predict potential targets of miR-497-5p (Figure 5A). It was discovered that three osteogenic-associated potential target genes including SMAD specific E3 ubiquitin protein ligase 1 (*Smurf1*), *Smurf2* and Smad nuclear interacting protein 1 (*SNIP1*), contain a miR-497b-5p binding site in their 3′-UTRs (Supplementary Figure 1). After miR-497-5p overexpressed or suppressed, three target genes were detected. The most significant change was observed in the mRNA and protein levels of *Smurf2* (Figures 5B,C). To further confirm whether miR-497-5p directly targets *Smurf2*, we constructed dual luciferase reporter vectors containing the Smurf2 3′UTR wild type (WT) or mutated sequence (Mut) (Figure 5D). The dual luciferase vector and miR-497-5p mimics (or mimics NC) were co-transfected into HEK 293T cells. Dual luciferase reporter assay showed that miR-497-5p dramatically suppressed the luciferase activity of WT Smurf2 than that of the negative control, and there was no significant alteration in the luciferase activity of mutated *Smurf2* (Figure 5E). The above demonstrated that miR-497-5p negatively regulated *Smurf2* by directly binding to the 3′UTR of its mRNA. In other words, *Smurf2* is a target gene of miR-497-5p.

### *Smurf2* Is Involved in Osteo/Odontogenic Differentiation of SCAP

**FIGURE 6:**
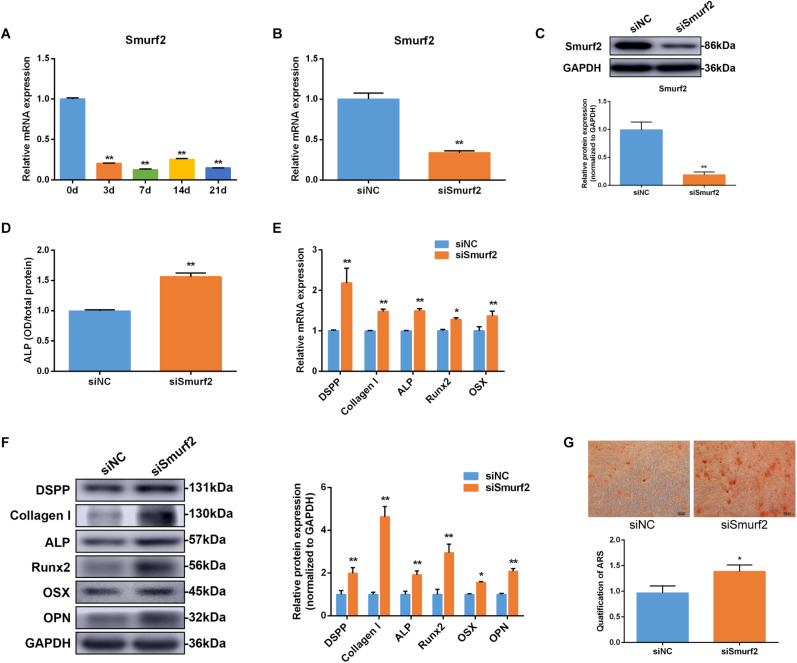
*Smurf2* was involved in osteo/odontogenic differentiation of SCAP. **(A)** *Smurf2* mRNA expression was examined via qRT-PCR at different time points during osteo/odontogenic differentiation of SCAP. **(B,C)** The knockdown efficiency of *Smurf2* siRNA was confirmed by qRT-PCR and western blot. **(D)** ALP activity was detected on day 7 after *Smurf2* knockdown. **(E,F)** mRNA and protein expression of osteo/odontogenic markers were examined by qRT-PCR and western blot after *Smurf2* knockdown. **(G)** Alizarin red staining and quantitative assay after *Smurf2* knockdown. Data are presented as mean ± SD of three independent experiments. *P < 0.05, **P < 0.01 compared with control group.

In this study, *Smurf2* mRNA expression decreased on day 3 and remained low level to day 21 during osteo/odontogenic differentiation of SCAP (Figure 6A). To confirm the effect of *Smurf2* on osteo/odontogenic differentiation of SCAP, *Smurf2* expression was interfered by transfecting siSmurf2 in SCAP. The knockdown efficiency was estimated by qRT-PCR and western blot. As our expected, the siSmurf2 could markedly decreased *Smurf2* expression (Figures 6B,C). Moreover, *Smurf2* knockdown significantly promoted the ALP activity and osteo/odontogenic genes expression (Figures 6D–F). The Alizarin red staining also confirmed that knockdown of *Smurf2* accelerated the osteogenic differentiation of SCAP (Figure 6G). These results demonstrated that *Smurf2* was involved in osteo/odontogenesis of SCAP.

### *Smurf2* Knockdown Blocked the Inhibitory Effect of miR-497-5p Inhibitor

**FIGURE 7:**
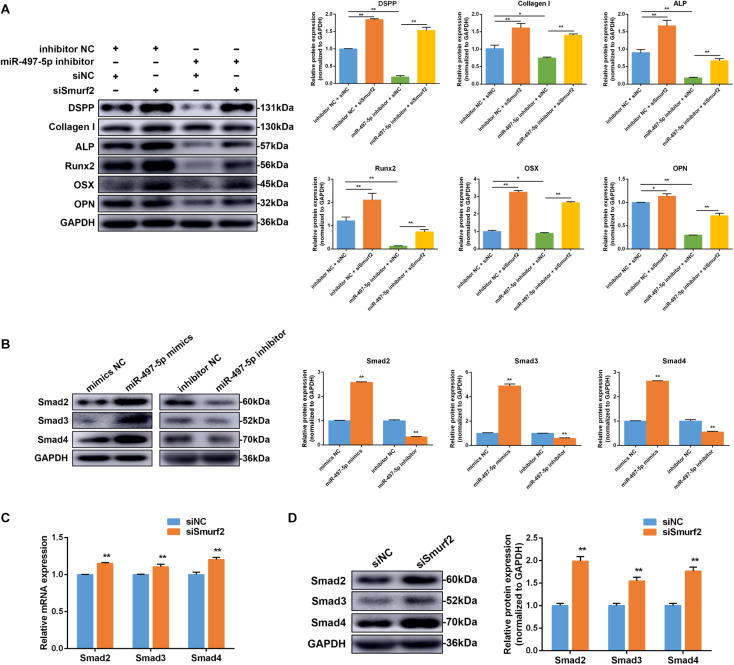
miR-497-5p modulated osteo/odontogenic differentiation of SCAP via Smad signaling pathway by targeting *Smurf2*. **(A)** The protein expression of osteo/odontogenic markers after co-transfected with miR-497-5p inhibitor and siSmurf2 or their respective negative controls. **(B)** Smad2, Smad3 and Smad4 protein expression level following miR-497-5p mimics and inhibitor transfection in SCAP. **(C,D)** *Smad2*, *Smad3*, and *Smad4* mRNA and protein levels examined via qRT-PCR and western blot following *Smurf2* knockdown in SCAP. Data are presented as mean ± SD of three independent experiments. *P < 0.05, **P < 0.01 compared with control group.

To further confirm whether the effect of miR-497-5p on osteo/odontogenic differentiation depended on *Smurf2* in SCAP, we co-transfected the miR-497-5p inhibitor and siSmurf2 or their respective negative controls into SCAP. As expected, the inhibitory effects of miR-497-5p inhibitor on SCAP were suppressed after Smurf2 knockdown (Figure 7A). The result indicated that miR-497-5p regulated osteo/odontogenic differentiation of SCAP through targeting *Smurf2*.

### MiR-497-5p Regulated Osteo/Odontogenic Differentiation in SCAP via the Smad Signaling Pathway

To determine if miR-497-5p increased SCAP osteo/odontogenic differentiation through the Smad signaling pathway, miR-497-5p mimics or inhibitor were transfected into cells. Western blot revealed Smad2, Smad3 and Smad4 expression levels were upregulated after transfection with miR-497-5p mimics and downregulated after transfection with miR-497-5p inhibitors (Figure 7B). The results indicated that Smad signaling pathway was involved in miR-497-5p-mediated osteo/odontogenic differentiation. Moreover, we further confirmed that mRNA and protein levels of Smad2, Smad3 and Smad4 were highly upregulated following transfection of siSmurf2 into SCAP (Figures 7C,D). All these results demonstrated that miR-497-5p promote osteo/odontogenic differentiation of SCAP via Smad signaling pathway by targeting *Smurf2*.

## Discussion

Recent studies indicated that miRNAs were involved in diverse biological processes including cell proliferation, differentiation, apoptosis, and carcinogenesis. MiR-497 was reported to be upregulated or downregulated in various cancers, thereby suggesting its diverse roles in different tissues (Yan et al., 2008; Lan et al., 2014; Maura et al., 2015). However, its role in osteogenic/odontogenic differentiation and related mechanisms require further investigation. In the present study, we first discovered miR-497-5p was upregulated during osteo/odontogenic differentiation in human SCAP and overexpression of miR-497-5p increased its osteo/odontogenic differentiation of SCAP. This is consistent with a previous study, which reported that overexpression of miR-497∼195 promotes CD31^*hi*^EMCN^*hi*^ endothelial cells angiogenesis and osteogenesis (Yang et al., 2017). Contrary to our findings, miR-497 suppressed proliferation and osteogenic differentiation in human primary mesenchymal stromal/stem cells (MSC) (Almeida et al., 2016). These results might be attributed to distinct origin of the cells utilized in several differentiation studies. Human MSC and SCAP, as postnatal mesenchymal stem cells, share some similarity, however, they have their own characteristics. The tissue-specific expression of miRNAs corresponded with the diversity of their functions. The SCAP of developing tooth root have their distinct biological characteristics (Sonoyama et al., 2006, 2008).

To investigate the molecular mechanism by which miR-497-5p promoted human SCAP osteo/odontogenic differentiation, we searched for potential target genes of miR-497-5p. We identified *Smurf2* was a direct target gene of miR-497-5p by using bioinformatics analysis and dual luciferase reporter assay. Smurf2 is a member of E3 ubiquitin ligases (Lin et al., 2000; Zhang et al., 2001). It interacted with Smad proteins (important proteins of TGF-β/Smad signaling pathway) and other osteogenic-related genes, leading to their degradation and thereby the negative regulation of the TGF-β/Smad signaling pathway and osteogenic differentiation process (Zhang et al., 2001; Izzi and Attisano, 2004; Kaneki et al., 2006; Lönn et al., 2008). Kaneki et al. (2006) found that tumor necrosis factor inhibited bone formation by promoting *Runx2* degradation via upregulation of *Smurf1* and *Smurf2 in vitro* and *in vivo*. The latest study showed that TRAF4 up-regulated the osteogenic differentiation of MSCs by acting as an E3 ubiquitin ligase to mediate the ubiquitination of Smurf2 and cause Smurf2 degradation (Li et al., 2019). In our study, overexpression of miR-497-5p significantly suppressed the expression of *Smurf2*. In contrast, inhibition of miR-497-5p increased *Smurf2* expression. More importantly, dual luciferase reporter gene assay confirmed that *Smurf2* was a direct target of miR-497-5p.

Despite it has been reported that *Smurf2* is the target gene of miR-497 in lung cancer cells (Dong-Kyu et al., 2019), miRNA profiles are tissue-specific. Specific cell type differentiation is a process involving complex network transcription factors. However, the regulation of miR-497-5p and *Smurf2* on osteo/odontogenic differentiation in SCAP is largely unknown. Therefore, we further investigated the effects of *Smurf2* on osteo/odontogenic differentiation in SCAP. The finding showed that *Smurf2* was downregulated during the odontogenic differentiation of SCAP and *Smurf2* knockdown significantly elevated expression of osteo/odontogenic markers, thus resembling the effect of miR-497-5p overexpression. A rescue effect was observed, wherein *Smurf2* knockdown could block the effect of miR-497-5p inhibition in SCAP osteo/odontogenic differentiation. In conclusion, our study demonstrated that miR-497-5p enhanced osteo/odontogenic differentiation in SCAP through suppressing *Smurf2* expression.

Smad signaling pathway plays a crucial role in osteogenic differentiation. The ubiquitin-proteasome degradation is a vital mechanism regulating TGF-β/Smad pathway. *Smurf2*, an important component of ubiquitin-proteasome system, participates in its regulation (Kavsak et al., 2000; Zhang et al., 2001; Ganji et al., 2015). The signal transduction of the TGF-β/Smad pathway mainly depends on the Smad proteins, which act as critical intracellular receptors, including Smad1-8 (Massagué and Wotton, 2000). For example, Smurf2 interacted with Smad7 to compose a complex in the nucleus, then entered the cytoplasm to degrade TβRI, and inhibits the signal transduction of TGF-β (Ganji et al., 2015). It also has been reported that Smurf2 combining Smad7-induced output and collection activated TGF-β receptors. Then the receptor and Smad7 were degraded via the proteasome and lysosomal pathways (Kavsak et al., 2000). In this study, we observed Smad2, Smad3 and Smad4 protein levels were upregulated by miR-497-5p mimics and decreased by miR-497-5p inhibitors, indicating Smad signaling pathway was activated by miR-497-5p. Furthermore, when *Smurf2* was knocked down by siRNA, *Smad2*, *Smad3* and *Smad4* mRNA and protein levels were upregulated. Our results suggested that miR-497-5p could directly target *Smurf2* and regulate the osteo/odontogenic differentiation through Smad signaling pathway.

In the future study, additional strategies might investigate the effect of miR-497-5p on the osteo/odontogenic differentiation under the undifferentiated condition and more studies could be performed to confirm these factors by well-designed *in vivo* experiments.

**FIGURE 8:**
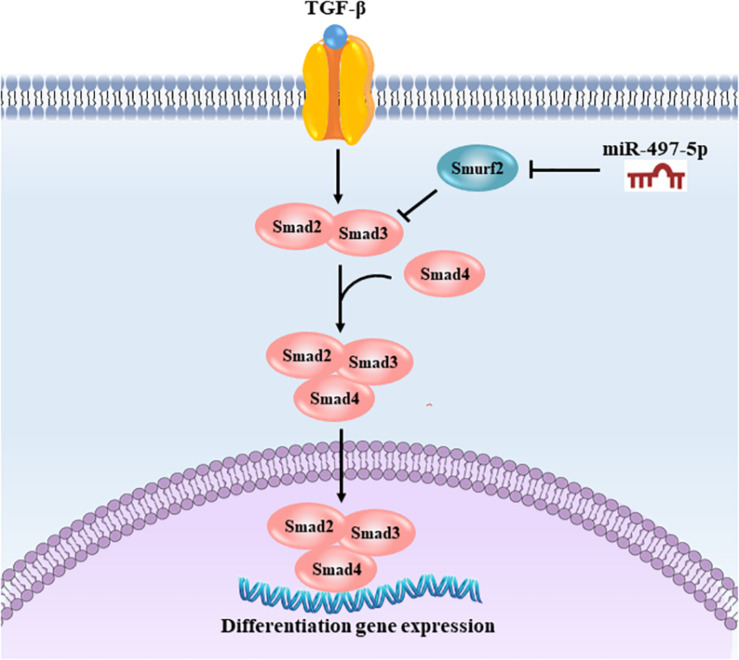
Schematic illustration of miR-497-5p regulating osteo/odontogenic differentiation of SCAP. miR-497-5p suppresses *Smurf2* expression, resulting in upregulating Smad signaling pathway, thereby promoting osteo/odontogenic differentiation.

In summary, our study was the first to demonstrate that miR-497-5p acted as a positive regulator of osteo/odontogenic differentiation in SCAP. Importantly, miR-497-5p promoted osteo/odontogenic differentiation by targeting *Smurf2* and modulating the Smad signaling pathway as depicted in Figure 8. Our findings reveal a novel function of miR-497-5p in the osteo/odontogenic differentiation, and suggest the application of miR-497-5p as a potential target to enhance dental pulp/dentine regeneration and to develop new therapeutic approaches in dental tissue regeneration.

## Data Availability Statement

The microarray data has been deposited in GEO: GSE154466.

## Ethics Statement

The studies involving human participants were reviewed and approved by Ethics Committee of the School and Hospital of Stomatology, Shandong University. Written informed consent to participate in this study was provided by the participants’ legal guardian/next of kin.

## Author Contributions

JL, XW, CZ, and YW contributed in conception and design. JL, XW, MS, and JD contributed in performing experiments. JL, JY, and WZ contributed in data collection, interpretation and statistical analysis. JL and XW contributed in drafting manuscript. CZ and YW contributed in critically revise manuscript. All authors read and approved the final manuscript.

## Conflict of Interest

The authors declare that the research was conducted in the absence of any commercial or financial relationships that could be construed as a potential conflict of interest.

## Permission to Reuse and Copyright

All authors approved the reuse and copyright licenses of this study.

## References

[B1] Abe S. Yamaguchi S. Watanabe A. Hamada K. Amagasa T. (2008). Hard tissue regeneration capacity of apical pulp derived cells (APDCs) from human tooth with immature apex. *Biochem. Biophys. Res. Commun*. 371 90–93. 10.1016/j.bbrc.2008.04.016 18407827

[B2] Almeida M. I. Silva A. M. Vasconcelos D. M. Almeida C. R. Caires H. Pinto M. T. (2016). miR-195 in human primary mesenchymal stromal/stem cells regulates proliferation, osteogenesis and paracrine effect on angiogenesis. *Oncotarget* 7 7–22. 10.18632/oncotarget.6589 26683705 PMC4807979

[B3] Bakopoulou A. Leyhausen G. Volk J. Tsiftsoglou A. Garefis P. Koidis P. (2011). Comparative analysis of in vitro osteo/odontogenic differentiation potential of human dental pulp stem cells (DPSCs) and stem cells from the apical papilla (SCAP). *Arch. Oral Biol*. 56 709–721. 10.1016/j.archoralbio.2010.12.008 21227403

[B4] Bartel D. P. (2004). MicroRNAs: genomics, biogenesis, mechanism, and function. *Cell* 116 281–297. 10.1016/S0092-8674(04)00045-5 14744438

[B5] Chen S. Zheng Y. Zhang S. Jia L. Zhou Y. (2017). Promotion effects of miR-375 on the osteogenic differentiation of human adipose-derived mesenchymal stem cells. *Stem Cell Rep*. 8 773–786. 10.1016/j.stemcr.2017.01.028 28262546 PMC5355733

[B6] Chrepa V. Austah O. Diogenes A. (2017). Evaluation of a commercially available hyaluronic acid Hydrogel (Restylane) as injectable scaffold for dental pulp regeneration: an in vitro evaluation. *J. Endod*. 43 257–262. 10.1016/j.joen.2016.10.026 28041686

[B7] Dong-Kyu C. Jinyoung P. Moonsoo C. Eunmi B. Mihue J. Young S. Y. (2019). MiR-195 and miR-497 suppress tumorigenesis in lung cancer by inhibiting SMURF2-induced TGF-β receptor I ubiquitination. *Mol. Oncol*. 13 2663–2678. 10.1002/1878-0261.12581 31581360 PMC6887584

[B8] Ganji A. Roshan H. M. Varasteh A. Moghadam M. Sankian M. (2015). The effects of WW2/WW3 domains of Smurf2 molecule on TGF-beta signaling and arginase I gene expression. *Cell Biol. Int*. 39 690–695. 10.1002/cbin.10446 25612247

[B9] Hammond S. M. Caudy A. A. Hannon G. J. (2001). Post-transcriptional gene silencing by double-stranded RNA. *Nat. Rev. Genet*. 2 110–119. 10.1038/35052556 11253050

[B10] Huang G. T. Yamaza T. Shea L. D. Djouad F. Kuhn N. Z. Tuan R. S. (2010). Stem/progenitor cell-mediated de novo regeneration of dental pulp with newly deposited continuous layer of dentin in an in vivo model. *Tissue Eng. Part A* 16 605–615. 10.1089/ten.TEA.2009.0518 19737072 PMC2813150

[B11] Izzi L. Attisano L. (2004). Regulation of the TGFbeta signalling pathway by ubiquitin-mediated degradation. *Oncogene* 23 2071–2078. 10.1038/sj.onc.1207412 15021894

[B12] Julia W. Stephanie J. Sarina K. Richard G. I Sven D. (2009). Many roads to maturity: microRNA biogenesis pathways and their regulation. *Nat. Cell Biol*. 11 228–234. 10.1038/ncb0309-228 19255566

[B13] Kaneki H. Guo R. Chen D. Yao Z. Schwarz E. M. Zhang Y. E. (2006). Tumor necrosis factor promotes Runx2 degradation through up-regulation of Smurf1 and Smurf2 in osteoblasts. *J. Biol. Chem*. 281 4326–4333. 10.1074/jbc.M509430200 16373342 PMC2647592

[B14] Kavsak P. Rasmussen R. K. Causing C. G. Bonni S. Zhu H. Thomsen G. H. (2000). Smad7 binds to Smurf2 to form an E3 ubiquitin ligase that targets the TGF beta receptor for degradation. *Mol. Cell* 6 1365–1375. 10.1016/S1097-2765(00)00134-9 11163210

[B15] Koutsoumparis A. Vassili A. Bakopoulou A. Ziouta A. Tsiftsoglou A. S. (2018). Erythropoietin (rhEPOa) promotes endothelial transdifferentiation of stem cells of the apical papilla (SCAP). *Arch. Oral Biol*. 96 96–103. 10.1016/j.archoralbio.2018.09.001 30205239

[B16] Lan J. Xue Y. Chen H. Zhao S. Wu Z. Fang J. (2014). Hypoxia-induced miR-497 decreases glioma cell sensitivity to TMZ by inhibiting apoptosis. *FEBS Lett*. 588 3333–3339. 10.1016/j.febslet.2014.07.021 25080009

[B17] Li J. Wang P. Xie Z. Wang S. Cen S. Li M. (2019). TRAF4 positively regulates the osteogenic differentiation of mesenchymal stem cells by acting as an E3 ubiquitin ligase to degrade Smurf2. *Cell Death Differ*. 26 2652–2666. 10.1038/s41418-019-0328-3 31076633 PMC7224386

[B18] Lin X. Liang M. Feng X. H. (2000). Smurf2 is a ubiquitin E3 ligase mediating proteasome-dependent degradation of Smad2 in transforming growth factor-beta signaling. *J. Biol. Chem*. 275 36818–36822. 10.1074/jbc.C000580200 11016919

[B19] Lindsay M. A. (2008). microRNAs and the immune response. *Trends Immunol*. 29 343–351. 10.1016/j.it.2008.04.004 18515182

[B20] Liu F. Wang X. Yang Y. Hu R. Wang W. Wang Y. (2019a). The suppressive effects of miR-508-5p on the odontogenic differentiation of human dental pulp stem cells by targeting glycoprotein non-metastatic melanomal protein B. *Stem Cell Res. Ther*. 10:35. 10.1186/s13287-019-1146-8 30670091 PMC6341723

[B21] Liu J. Du J. Chen X. Yang L. Zhao W. Song M. (2019b). The effects of mitogen-activated protein kinase signaling pathways on lipopolysaccharide-mediated osteo/odontogenic differentiation of stem cells from the apical papilla. *J. Endod*. 45 161–167. 10.1016/j.joen.2018.10.009 30711172

[B22] Lönn P. Morén A. Raja E. Dahl M. Moustakas A. (2008). Regulating the stability of TGFβ receptors and Smads. *Cell Res*. 19 21–35. 10.1038/cr.2008.308 19030025

[B23] Massagué J. Wotton D. (2000). Transcriptional control by the TGF-β/Smad signaling system. *EMBO J*. 19 1745–1754. 10.1093/emboj/19.8.1745 10775259 PMC302010

[B24] Maura F. Cutrona G. Mosca L. Matis S. Lionetti M. Fabris S. (2015). Association between gene and miRNA expression profiles and stereotyped subset #4 B-cell receptor in chronic lymphocytic leukemia. *Leuk. Lymphoma* 56 3150–3158. 10.3109/10428194.2015.1028051 25860243

[B25] Na S. Zhang H. Huang F. Wang W. Ding Y. Li D. (2016). Regeneration of dental pulp/dentine complex with a three-dimensional and scaffold-free stem-cell sheet-derived pellet. *J. Tissue Eng. Regen. Med*. 10 261–270. 10.1002/term.1686 23365018

[B26] Ritchie M. E. Phipson B. Wu D. Hu Y. Law C. W. Shi W. (2015). Limma powers differential expression analyses for RNA-sequencing and microarray studies. *Nucleic Acids Res.* 43:e47. 10.1093/nar/gkv007 25605792 PMC4402510

[B27] Sonoyama W. Liu Y. Fang D. Yamaza T. Seo B.-M. Zhang C. (2006). Mesenchymal stem cell-mediated functional tooth regeneration in swine. *PLoS One* 1:e79. 10.1371/journal.pone.0000079 17183711 PMC1762318

[B28] Sonoyama W. Liu Y. Yamaza T. Tuan R. S. Wang S. Shi S. (2008). Characterization of the apical papilla and its residing stem cells from human immature permanent teeth: a pilot study. *J. Endod.* 34 166–171. 10.1016/j.joen.2007.11.021 18215674 PMC2714367

[B29] Sun F. Wan M. Xu X. Gao B. Zhou Y. Sun J. (2014). Crosstalk between miR-34a and Notch signaling promotes differentiation in apical papilla stem cells (SCAPs). *J. Dent. Res*. 93 589–595. 10.1177/0022034514531146 24710391

[B30] Tang J. Zhang Z. Jin X. Shi H. (2018). miR-383 negatively regulates osteoblastic differentiation of bone marrow mesenchymal stem cells in rats by targeting Satb2. *Bone* 114 137–143. 10.1016/j.bone.2018.06.010 29909059

[B31] Wan F. Gao L. Lu Y. Ma H. Wang H. Liang X. (2016). Proliferation and osteo/odontogenic differentiation of stem cells from apical papilla regulated by Zinc fingers and homeoboxes 2: an in vitro study. *Biochem. Biophys. Res. Commun*. 469 599–605. 10.1016/j.bbrc.2015.11.135 26679602

[B32] Wang W. Wang X. Li C. Chen T. Zhang N. Liang Y. (2019). Huaier suppresses breast cancer progression via linc00339/miR-4656/CSNK2B signaling pathway. *Front. Oncol.* 9:1195. 10.3389/fonc.2019.01195 31781497 PMC6857111

[B33] Yan L. X. Huang X. F. Shao Q. Huang M. Y. Deng L. Wu Q. L. (2008). MicroRNA miR-21 overexpression in human breast cancer is associated with advanced clinical stage, lymph node metastasis and patient poor prognosis. *RNA* 14 2348–2360. 10.1261/rna.1034808 18812439 PMC2578865

[B34] Yang H. Ma L. Han X. Yang L. Tian C. Wang Y. (2012). The effects of tumor necrosis factor-alpha on mineralization of human dental apical papilla cells. *J. Endod*. 38 960–964. 10.1016/j.joen.2012.04.005 22703661

[B35] Yang M. Li C. J. Sun X. Guo Q. Xiao Y. Su T. (2017). MiR-497 approximately 195 cluster regulates angiogenesis during coupling with osteogenesis by maintaining endothelial Notch and HIF-1alpha activity. *Nat. Commun*. 8:16003. 10.1038/ncomms16003 28685750 PMC5504303

[B36] Yao S. Zhao W. Ou Q. Liang L. Lin X. Wang Y. (2017). MicroRNA-214 suppresses osteogenic differentiation of human periodontal ligament stem cells by targeting ATF4. *Stem Cells Int*. 2017:3028647. 10.1155/2017/3028647 29213288 PMC5682087

[B37] Zhang Y. Chang C. Gehling D. J. Hemmati-Brivanlou A. Derynck R. (2001). Regulation of Smad degradation and activity by Smurf2, an E3 ubiquitin ligase. *Proc. Natl. Acad Sci. U.S.A*. 98 974–979. 10.1073/pnas.98.3.974 11158580 PMC14694

