# Supplementary material for: MiR-497-5p Regulates Osteo/Odontogenic Differentiation of Stem Cells From Apical Papilla via the Smad Signaling Pathway by Targeting Smurf2
Source: Front Genet. 2020 Oct 30;11:582366. doi: 10.3389/fgene.2020.582366 (PMC7662069; doi:10.3389/fgene.2020.582366)
Supplement: Supplementary file 1 [file Data_Sheet_1.docx]

Supplementary Material

# TABLE 1

**TABLE 1** Primers used for quantitative real-time PCR.

| Primer name | Sequences（5’-3’） |
| --- | --- |
| DSPP | F: GCATTTGGGCAGTAGCATGG  R: CTGACACATTTGATCTTGCTAGGAG |
| Collagen I | F: GCTGATGATGCCAATGTGGTT  R: CCAGTCAGAGTGGCACATCTTG |
| ALP | F: CCACGTCTTCACATTTGGTG  R: AGACTGCGCCTGGTAGTTGT |
| Runx2 | F: TCCACACCATTAGGGACCATC  R: TGCTAATGCTTCGTGTTTCCA |
| BSP | F: CTGGCACAGGGTATACAGGGTTAG  R: GCCTCTGTGCTGTTGGTACTGGT |
| OSX | F: CCTGCTTGAGGAGGAAGTTCACTAT  R: GAGTACGGCTTCTTTGTGCC |
| SNIP1 | F: AAGAAGCAAGTCTCCTCGCAG  R: GTTCTGATGGTTCCCTGTGCT |
| Smurf1 | F: GTCCAGAAGCTGAAAGTCCTCAGA  R: CACGGAATTTCACCATCAGCC |
| Smurf2 | F: TGGATCAGGAAGTCGGAAAA  R: GGACATGTCTAACCCCGGA |
| GAPDH | F: GCACCGTCAAGGCTGAGAAC  R: TGGTGAAGACGCCAGTGGA |
| hsa-miR-376a-3p | F: TACCCATTGCATATCGGAGTTG |
| hsa-miR-497-5p | F: CAGCAGCACACTGTGGTTTGT |
| hsa-miR-660-5p | F: ACCACTGACCGTTGACTGTACC |
| hsa-miR-146a-5p | F: CATAGCCCGGTCGCTGGTACATGA |
| hsa-miR-199b-5p | F: CCTCACCATCCCTTCTGCCTGC |
| all miRNA reverse primer | Universal adaptor primer |
| U6 | F: GGAACGATACAGAGAAGATTAGC  R: TGGAACGCTTCACGAATTTGCG |

# FIGURE 1


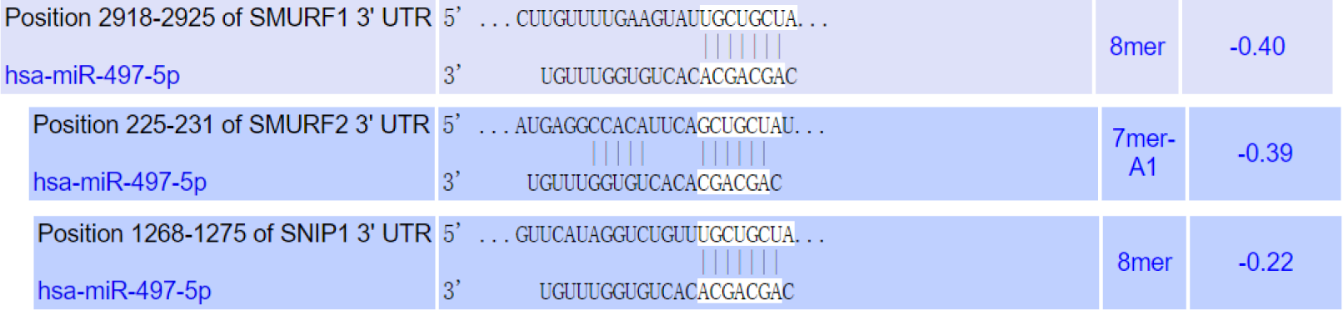


**FIGURE 1** MiR-497-5p binding site in the 3’-UTRs of Smurf1, Smurf2 and SNIP1.
